# Effects of *Lactobacillus salivarius*-containing tablets on caries risk factors: a randomized open-label clinical trial

**DOI:** 10.1186/1472-6831-14-110

**Published:** 2014-09-02

**Authors:** Tetsuyo Nishihara, Nao Suzuki, Masahiro Yoneda, Takao Hirofuji

**Affiliations:** 1 Section of General Dentistry, Department of General Dentistry, Fukuoka Dental College, 2-15-1 Tamura, Sawara-ku, Fukuoka, 801-0193, Japan

**Keywords:** Caries risk factor, *Lactobacillus salivarius*, Mutans streptococci, Oral environment, Probiotics

## Abstract

**Background:**

To evaluate the effects of the lactic acid bacterium *Lactobacillus salivarius* on caries risk factors.

**Methods:**

The study was performed in 64 healthy volunteers to evaluate the effects of *L. salivarius*-containing tablets on caries risk factors. The participants were divided randomly into four groups, and took tablets containing *L. salivarius* WB21, *L. salivarius* TI 2711, Ovalgen® DC (antibody against glucosyltransferase from *Streptococcus mutans*), or xylitol. Levels of mutans streptococci and lactobacilli, amount of salivary flow, salivary pH, and salivary buffering capacity were assessed before and after taking the tablets. Subsequently, a short-term administration trial using *L. salivarius* WB21-containing tablets was performed in eight healthy volunteers. The participants took *L. salivarius* WB21-containing tablets (2.0 × 10^9^ colony forming units/day) for 2 weeks, and the numbers of mutans streptococci in saliva were counted.

**Results:**

The levels of mutans streptococci seemed to decrease in the *L. salivarius* WB21, TI 2711, and Ovalgen® DC groups compared to the xylitol group, with no significant differences between the groups. Lactobacilli levels significantly increased in the *L. salivarius* WB21 and TI 2711 groups compared to the other groups. Concerning salivary flow and salivary pH, no significant differences were observed between the groups. The salivary buffering capacity significantly increased in the *L. salivarius* TI 2711 group (*P* = 0.003) and Ovalgen® DC group (*P* = 0.002) compared to the xylitol group. The short-term administration trial showed that the *L. salivarius* WB21-containing tablets significantly decreased the number of mutans streptococci (*P* = 0.039).

**Conclusion:**

*L. salivarius*-containing tablets were suggested to increase resistance to caries risk factors.

**Trial registration:**

UMIN000013160 (registration date: February 14, 2014).

## Background

Beneficial microorganisms, or probiotics, have various functions including antibacterial activities [1], modulation of the host immune response [2], antiallergic effects [3], and cancer prevention in the human intestine [4]. A number of studies using lactic acid bacteria for the prevention of oral diseases have been reported [5-10]. Probiotic bacteria in the human oral cavity include *Bifidobacterium* species and *Lactobacillus* species [11]. Oral administration of *Lactobacillus salivarius* WB21-containing tablets and oil reduced plaque accumulation, periodontal pocket depth, bleeding on probing, and oral malodor [7-10]. *L. salivarius* TI 2711 showed antibacterial activity against *Porphyromonas gingivalis* in mixed culture experiments [12], and a clinical trial indicated that the number of *P. gingivalis* in subgingival plaque was reduced by oral administration of *L. salivarius* TI 2711-containing tablets but recovered following cessation of tablet administration [13]. However, there have been no reports regarding caries prevention and control by *L. salivarius*.

*Lactobacillus* species are microbial markers of dental caries risk [14]. Some species of lactobacilli have been reported to occur in large numbers in both superficial and deep caries [15,16]. However, several *Lactobacillus* species have been isolated from healthy mouths [17,18]. Increased production of organic acids in the dental plaque would be considered a side effect of probiotic lactobacilli. Most studies of caries prevention and control by lactic acid bacteria were performed over the last few years. *Lactobacillus* species, including *Lactobacillus rhamnosus*, *Lactobacillus reuteri*, and *Lactobacillus paracasei*, that were investigated in previous clinical trials did not alter or reduce mutans streptococci levels [19-21]. No adverse effects or potential risks of these bacteria were reported. In the present study, the potential effects of tablets containing *L. salivarius* WB21 or TI 2711 on caries risk factors were compared with tablets containing an antibody against *Streptococcus mutans* and those containing only xylitol as controls. In addition, oral administration of *L. salivarius* WB21-containing tablets for 2 weeks was performed to evaluate their effect on the levels of mutans streptococci.

## Methods

### Products

The tablets used in this study are listed in Table 1. The tablets containing *L. salivarius* WB21 (Minna No Zendamakin WB21 Tablet®; Wakamoto Pharmaceutical Co., Tokyo, Japan) contained 6.7 × 10^8^ colony-forming units (CFU) of *L. salivarius* WB21 and 280 mg of xylitol per tablet. The tablets containing *L. salivarius* TI 2711 (Super Kurish; Frente International Co., Tokyo, Japan) contained 2.8 × 10^8^ CFU of *L. salivarius* TI 2711 and 450 mg of xylitol per tablet. The Ovalgen® DC-containing tablets (Hakira; BeanStalk, Tokyo, Japan) contained egg yolk antibodies against glucosyltransferase of *S. mutans* and 100 mg of xylitol per tablet. The xylitol-containing tablets contained 280 mg of xylitol per tablet (Wakamoto Pharmaceutical Co.).

**Table 1:** **Study population and products used in the study [*n *** **(%) or median (IQR)]**

**Group ( *n *)**	***L. salivarius *** **WB21**	***L. salivarius *** **TI 2711**	**Ovalgen® DC**	**Xylitol**
**Item**	**(*n*** **= 17)**	**(*n*** **= 16)**	**(*n*** **= 13)**	**(*n*** **= 18)**
Age	24.0 (23.0 to 25.0)	24.0 (23.0 to 25.0)	24.0 (23.0 to 25.0)	24.5 (23.0 to 26.0)
Female (%)	9 (52.9)	6 (37.5)	4 (30.8)	6 (33.3)
Mutans streptococci level (%)				
Class 0	10 (58.8)	10 (62.5)	2 (15.4)	9 (50.0)
Class 1	2 (11.8)	2 (12.5)	4 (30.8)	5 (27.8)
Class 2	3 (17.6)	2 (12.5)	3 (23.1)	2 (11.1)
Class 3	2 (11.8)	2 (12.5)	4 (30.8)	2 (11.1)
Lactobacilli level (%)				
Class 0	5 (62.5)	6 (75.0)	4 (57.1)	5 (71.4)
Class 1	0 (0.0)	1 (12.5))	1 (14.3)	2 (28.6)
Class 2	3 (37.5)	1 (12.5)	2 (28.6)	0 (0.0)
Class 3	0 (0.0)	0 (0.0)	0 (0.0)	0 (0.0)
Salivary flow (mL per 3 min)	4.8 (3.3 to 5.8)	7.3 (5.8 to 8.8)	6.5 (3.5 to 7.0)	5.5 (3.6 to 7.0)
Salivary pH	7.7 (7.6 to 7.9)	7.9 (7.7 to 7.9)	7.7 (7.5 to 7.9)	7.7 (7.6 to 8.0)
Buffering capacity	5.7 (5.2 to 6.1)	6.1 (5.9 to 6.1)	5.9 (5.2 to 6.2)	5.9 (5.6 to 6.3)
Product name (company)	Minna No Zendamakin WB21 Tablet (Wakamoto Pharmaceutical Co.)	Super Kurish (Frente International Co.)	Hakira (BeanStalk)	Xylitol tablets (not for sale) (Wakamoto Pharmaceutical Co.)
Active ingredient (per tablet)	*L. salivarius* WB21: 6.7 × 10^8^ CFU	*L. salivarius* TI 2711: 2.8 × 10^8^ CFU	Ovalgen® DC	Xylitol: 280 mg
	Xylitol: 280 mg	Xylitol: 450 mg	Xylitol: 100 mg	
Flavor	Mint	Clean mint	Orange	Mint

### Study population

Sixth-year dental students at Fukuoka Dental College were recruited for an open-label comparative trial, and 64 volunteers (25 females and 39 males; mean age, 24.8 ± 2.3 years) were included in the study. Short-term administration of *L. salivarius* WB21-containing tablets was performed in eight healthy volunteers (four females and four males, mean age, 30.0 ± 5.2 years), all of whom were dentists working at Fukuoka Dental College Medical and Dental Hospital, Fukuoka, Japan. The eligibility criteria were as follows: not currently visiting a dentist for treatment, no antibiotic use within 3 months, and no adverse reactions to lactose or fermented milk products. All subjects understood the nature of the research project and provided written, informed consent prior to enrollment. Permission for the study was obtained from the Ethics Committee for Clinical Research of Fukuoka Dental College and Fukuoka College of Health Sciences (approval no. 221).

### Study design

#### Open-label comparative trial

The trial was carried out from May to June in 2013 at Fukuoka Dental College Medical and Dental Hospital, Fukuoka, Japan. The participants were randomly divided into four groups in a lottery, and took *L. salivarius* WB21-containing tablets (*n* = 17), *L. salivarius* TI 2711-containing tablets (*n* = 16), Ovalgen® DC-containing tablets (*n* = 13), or xylitol-containing tablets (*n* = 18) (Table 1). They were directed to place a tablet on the tongue for a few minutes and allow it to dissolve. Levels of mutans streptococci and lactobacilli, amount of salivary flow, salivary pH, and salivary buffering capacity were assessed before and after taking the tablets.

The level of mutans streptococci was evaluated using Dentocult® SM Strip mutans (Orion Diagnostica, Espoo, Finland). First, the participants chewed paraffin gum for 3 min to stimulate the secretion of saliva and to transfer mutans streptococci from tooth surfaces into the saliva. Next, they pressed the test strip against the saliva on the tongue. The test strip was attached to the cap in the selective culture broth and the vial was recapped. After incubation at 37°C for 48 h, the results were classified into four classes (0–3) according to the model chart. The colony counts of mutans streptococci were recorded if possible. Table 2 shows the criteria used to evaluate changes in mutans streptococci level before and after taking the tablets.

**Table 2:** Criteria for evaluating changes in levels of mutans streptococci and lactobacilli

**Score**	**Criterion**
−3	Going down more than two classes
−2	Going down a class
−1	Same class and decrease in colony numbers
0	No change
+1	Same class and increase in colony numbers
+2	Going up a class
+3	Going up more than two classes

Note: As an example, a score of “+2” indicates that the class of Dentocult® SM increased one level after taking a tablet compared with the level before taking a tablet.

The level of lactobacilli was evaluated using Dentocult® LB (Orion Diagnostica) in half of the participants in each group. One milliliter of saliva collected by the gum test was applied to the culture medium and cultivated at 37°C for 96 h. Changes in lactobacillus level before and after taking the tablets were evaluated according to the same criteria using Dentocult® SM (Table 2).

Salivary pH values both before and after addition of acid (to assess salivary buffer capacity) were investigated in the remaining saliva (CheckBuf test kit; Morita, Osaka, Japan). The amount of salivary flow was assessed by the gum test.

### Short-term administration trial

The trial was carried out from February to March 2014. The participants were asked to take *L. salivarius* WB21-containing tablets for 2 weeks. The dose throughout the test period was maintained at one tablet three times per day, taken orally, after eating and mouth cleaning. Participants were directed to place a tablet on the tongue for a few minutes and allow it dissolve. They were also instructed not to change their oral hygiene regimens and not to take other probiotic products throughout the study period. Neither professional prophylaxis nor tooth-brushing instruction was performed before or during the experimental period. Collecting saliva was performed at least 4 h after eating lunch. The selective medium for mutans streptococci was Mitis Salivarius Agar (Difco, Tokyo, Japan) supplemented with 20% sucrose and bacitracin (0.2 units/mL) (Sigma, St. Louis, MO) [22]. Stimulated saliva was collected by chewing gum for 3 min and diluted, and the number of colonies was calculated after anaerobic incubation at 37°C for 48 h.

### Statistical analysis

The Kruskal-Wallis test was to test for differences among the four groups in the open-label comparative trial. The Wilcoxon rank-sum test was used to compare pairs of groups in the open-label comparative trial. The Wilcoxon signed-rank test was used to evaluate the difference in mutans streptococci level between day 0 and day 14 in the short-term administration trial with *L. salivarius* WB21-containing tablets. Statistical analyses were conducted using the R software package, version 3.0.1 [23]. In all analyses, *P* < 0.05 was taken to indicate statistical significance.

## Results

### Open-label comparative trial

Baseline data for the study population are shown in Table 1. Mutans streptococci were detected in 69% of the study population (44/64) at baseline by Dentocult® SM. Although the mutans streptococci level in the Ovalgen® DC group seemed to be different from that in the other groups, no statistically significant differences were noted in mutans streptococci levels at baseline among the groups.

Changes in the levels of mutans streptococci and lactobacilli, salivary flow, salivary pH, and salivary buffering capacity after taking the tablet are shown in Table 3. No significant difference was detected between the changes in mutans streptococci levels among the four groups. However, the levels of mutans streptococci seemed to decrease in the *L. salivarius* WB21, TI 2711, and Ovalgen® DC groups [median 0.0 (IQR −0.1 to 0.0), 0.0 (0.0 to 1.3), and 0.0 (−1.0 to 1.0), respectively] compared to the xylitol group [0.0 (0.0 to 2.0)].

**Table 3:** Changes in levels of mutans streptococci and lactobacilli, salivary flow, salivary pH, and salivary buffering capacity after taking the tablet [median (IQR)]

**Group ( *n *)**	***L. salivarius *** **WB21**	***L. salivarius *** **TI 2711**	**Ovalgen® DC**	**Xylitol**	***P *** value*
**Items**	**(*n*** **= 17)**	**(*n*** **= 16)**	**(*n*** **= 13)**	**(*n*** **= 18)**	
Mutans streptococci level	0.0 (−1.0 to 0.0)	0.0 (0.0 to 1.3)	0.0 (−1.0 to 1.0)	0.0 (0.0 to 2.0)	0.214
Lactobacilli level	3.0 (2.0 to 3.0)	2.5 (2.0 to 3.0)	0.0 (0.0 to 0.5)	0.0 (−0.3 to 0.0)	<0.001†
Salivary flow (mL per 3 min)	0.0 (−0.5 to 0.0)	0.0 (−0.4 to 0.5)	−0.5 (−1.0 to 0.0)	−0.5 (−1.1 to 0.0)	0.172
Salivary pH	0.0 (0.0 to 0.2)	0.2 (0.0 to 0.3)	0.1 (0.0 to 0.2)	0.0 (−0.1 to 0.3)	0.715
Buffering capacity	0.0 (−0.4 to 0.3)	0.0 (−0.2 to 0.4)	0.2 (0.0 to 0.3)	−0.3 (−0.6 to −0.1)	0.004§

*The Kruskal-Wallis test was used to evaluate the differences among the four groups.

†Significantly different pairs: *L. salivarius* WB21 vs. Ovalgen® DC, *P* = 0.001; *L. salivarius* WB21 vs. Xylitol, *P* = 0.001; *L. salivarius* TI 2711 vs. Ovalgen® DC, *P* = 0.007; and *L. salivarius* TI 2711 vs. Xylitol, *P* = 0.002 (Wilcoxon rank-sum test).

§Significantly different pairs: *L. salivarius* TI 2711 vs. Xylitol, *P* = 0.003; and Ovalgen® DC vs. Xylitol, *P* = 0.002 (Wilcoxon rank-sum test).

A significant difference was observed in the levels of lactobacilli among the four groups (*P* < 0.001). The levels of lactobacilli increased markedly in both the *L. salivarius* WB21 and TI 2711 groups [median 3.0 (IQR 2.0 to 3.0) and 2.5 (2.0 to 3.0), respectively], and were significantly higher than those in the other two groups [0.0 (0.0 to 0.5) and 0.0 (−0.3 to 0.0), respectively]. The presence of viable lactobacilli in these tablets was confirmed.

No significant differences were observed in salivary flow and salivary pH among the four groups. However, salivary flow (mL per 3 min) tended to decrease in the Ovalgen® DC and xylitol groups [median −0.5 (IQR −1.0 to 0.0) and −0.5 (−1.1 to 0.0), respectively], but not in the *L. salivarius* WB21 and *L. salivarius* TI 2711 groups [0.0 (−0.5 to 0.0) and 0.0 (−0.4 to 0.5), respectively]. Salivary pH tended to increase in the *L. salivarius* TI 2711 and Ovalgen® DC groups [median 0.2 (IQR 0.0 to 0.3) and 0.1 (0.0 – 0.2), respectively].

Significant differences were detected in salivary buffering capacity among the four groups (*P* = 0.004). The salivary buffering capacity decreased in the xylitol group [median −0.3 (IQR −0.6 to −0.1)], but increased in the Ovalgen® DC group [0.2 (0.0 to 0.3)], while no changes were observed in the salivary buffering capacity in the *L. salivarius* WB21 and TI 2711 groups [median 0.0 (IQR −0.4 to 0.3) and 0.0 (−0.2 to 0.4), respectively]. Comparative analysis showed that there were significant differences between the xylitol group and the *L. salivarius* TI 2711 and the Ovalgen® DC groups (*P* = 0.003 and *P* = 0.002, respectively).

### Short-term administration trial

**Figure 1:**
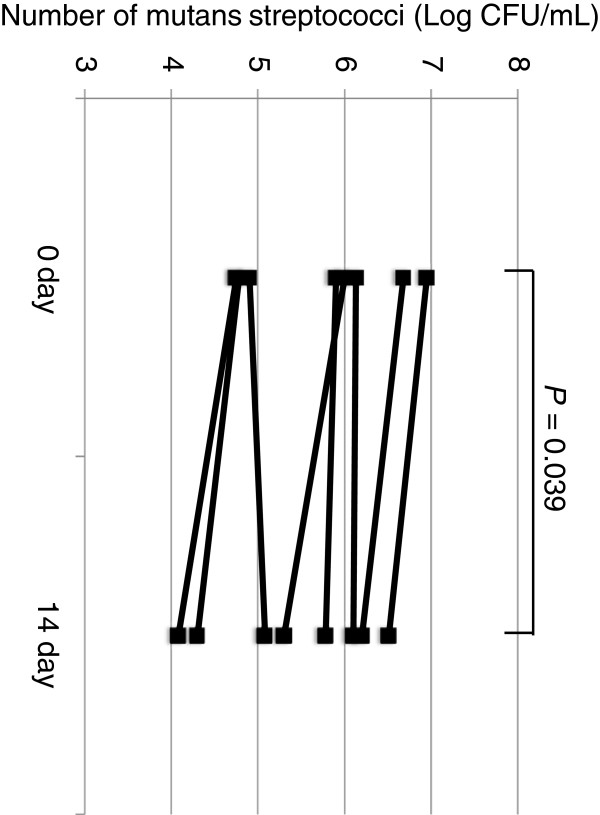
**Changes in the numbers of mutans streptococci (log CFU/mL) caused by continuous oral administration of *L. salivarius *WB21-containing tablets for 2 weeks.** A significant difference between day 0 and day 14 was shown by the Wilcoxon signed-rank test (*P* = 0.039).

The levels of mutans streptococci had decreased after 2 weeks of oral administration of *L. salivarius* WB21-containing tablets in all but one subject; there was a significant difference between days 0 and 14 [6.0 (4.9 to 6.3) to 5.5 (4.8 to 6.1) log CFU/mL; *P* = 0.039; Figure 1].

## Discussion

Oral consumption of *L. salivarius* WB21 and TI 2711 did not show side effects related to advance of caries in the oral cavity. In fact, those tablets provided better resistance to caries risk factors compared to the xylitol tablet. Mutans streptococci levels increased and salivary flow and salivary buffering capacity decreased after taking the xylitol tablet in the present study. In contrast, the mutans streptococci level decreased in the group taking oral *L. salivarius* WB21-containing tablets. A reduction in mutans streptococci level was seen in 29.4% of subjects in the *L. salivarius* WB21 group (5/17), 18.8% in the *L. salivarius* TI 2711 group (3/16), 38.5% in the Ovalgen® DC group (5/13), and 16.7% in the xylitol group (3/18). An increase in mutans streptococci level occurred in 11.8% of subjects in the *L. salivarius* WB21 group (2/17), 31.3% in the *L. salivarius* TI 2711 group (5/16), 30.8% in the Ovalgen® DC group (4/13), and 44.4% in the xylitol group (8/18). In addition, the exploratory short-term administration trial with *L. salivarius* WB21-containing tablets showed a reduction in levels of mutans streptococci in saliva at 2 weeks.

Salivary flow, salivary pH, and salivary buffering capacity did not change or increased after taking the *L. salivarius* WB21 and TI 2711 tablets. No changes were noted in salivary buffering capacity in the *L. salivarius* WB21 and TI 2711 groups, although a reduction was seen in the xylitol group. Medians salivary buffering capacity was the same in the *L. salivarius* WB21 and TI 2711 groups, but less variability was observed in the *L. salivarius* TI 2711 group than in the *L. salivarius* WB21 group (Table 3). A significant difference was detected in buffering capacity between the *L. salivarius* TI 2711 group and the xylitol group.

Salivary flow did not differ among the four groups in the present study, although it increased in the *L. salivarius* TI 2711 group [from 7.3 (5.8 to 8.8) to 8.0 (6.1 to 9.3) mL per 3 min]. The trend toward increased salivary pH in the *L. salivarius* TI 2711 group may have been caused by the increase in salivary flow (Table 3). No changes in salivary flow, salivary pH, and salivary buffering capacity were noted in the *L. salivarius* WB21 group. In previous studies, salivary flow was significantly increased by oral administration of *L. salivarius* WB21-containing products for 2 weeks [9,24]. Salivary pH and buffering capacity did not change in those studies. The increase in saliva in those studies may have been caused by continuous ingestion of the tablets.

Our results suggest that *L. salivarius* strain-containing tablets can be used safely without increasing the caries risk, and that their oral administration may contribute to caries control in healthy adults. An open-label trial is insufficient to confirm a positive effect of lactobacilli on caries prevention because placebo effects were evident in previous double-blind trials [7,9,10]. Therefore, a double-blind randomized placebo-controlled trial is required to confirm the effect of *L. salivarius*-containing tablets on caries risk factors.

Tablets containing egg yolk antibodies against *S. mutans* cell-associated glucosyltransferase (anti-CA-gtf IgY, Ovalgen® DC) and xylitol tablets were used for comparison in the present study. All products used contained xylitol, a non-fermentable sugar alcohol that cannot be used as an energy source in the metabolism of cariogenic bacteria [24]. In this study, the level of mutans streptococci in the saliva increased in 44.4% of subjects in the xylitol group. The gum test was performed twice, and therefore more mutans streptococci may have been removed from the surface of teeth. The flow rate, pH, and buffering capacity of saliva were reduced or remained unchanged by oral consumption of the xylitol tablets. A clinical trial using xylitol gum found that the effects of chewing are essential for the stimulation of salivary flow and the resulting recovery of pH levels and reduction of *S. mutans* levels in saliva [25]. It was suggested that licking a xylitol tablet does not affect the quality of saliva.

The Ovalgen® DC tablet significantly increased salivary buffering capacity compared with the xylitol tablet, although the cause is unclear. The rate of reduction in mutans streptococci levels in the Ovalgen DC® group (38.5%) was greater compared with the xylitol group (16.7%), although the differences between the groups were not significant. It has been reported that anti-CA-gtf IgY suppressed the development of caries lesions in rats [26], and tablets containing anti-CA-gtf IgY significantly reduced the numbers of mutans streptococci in a 5-day double-blind placebo-controlled trial [27]. The mean anaerobic bacterial counts were not significantly different before and after the trial [27], indicating that Ovalgen® DC is specific for dental cariogenic species. In contrast, lactic acid bacteria affect various bacteria residing in the oral cavity and have various functions that differ among species, including aggregation, competition for adhesion sites, nutrients, and growth factors, production of antimicrobial compounds such as acid, hydrogen peroxide, and bacteriocins, and effects on the immune response [28]. The mechanisms by which *L. salivarius* WB21 and TI 2711 reduce caries risks are unclear, but coaggregation, growth inhibition of mutans streptococci, and reduced plaque acidogenicity have been reported as potential mechanisms by which lactic acid bacteria prevent dental caries [29-31]. Keller et al. [29] investigated the *in vitro* abilities of eight probiotic *Lactobacillus* strains (*L. plantarum* 299v, 931, *L. rhamnosus* GG, LB21, *L. paracasei* F19, *L. reuteri* ATCC PTA 5289, DSM 17938 and *L. acidophilus* La5) to coaggregate and inhibit mutans streptococci. All lactobacilli displayed coaggregation activity and inhibited the growth of clinical mutans streptococci. The growth inhibition was strain-specific and dependent on both pH and cell density [29]. An *in vitro* experiment also indicated that lactic acid production in suspensions of plaque and probiotic lactobacilli was strain-dependent [32]. The salivary pH was not changed by oral consumption of *L. salivarius* WB21 or TI 2711 in this study. A previous clinical study using oil containing *L. salivarius* WB21 indicated no changes in salivary pH after 2 weeks of treatment [9]. The short-term consumption of *L. rhamnosus* GG and *L. reuteri* for 2 weeks had no effect on acid production by supragingival plaque [33]. In contrast, a significant reduction in plaque acidogenicity was found with long-term (6 weeks) consumption of *L. brevis* CD2 [31]. Furthermore, clinical assessments indicated that the effects of probiotics varied depending on host susceptibility. In fact, the levels of mutans streptococci in several participants were increased after oral consumption of *L. salivarius* strains in the present study. Further studies of the characteristics of probiotic strains and the host responses are required to determine their appropriate applications, doses and treatment durations.

## Conclusion

In conclusion, our results indicate that administration of tablets containing the probiotic lactic acid bacteria *L. salivarius* WB21 and TI 2711 for plaque control, periodontal health, and oral malodor is safe and helps to prevent the development of dental caries.

## Competing interests

The authors declare that they have no competing interest.

## Authors’ contributions

SN designed the study, analyzed the data, and wrote the manuscript. NT collected salivary samples and carried out the bacterial and salivary experiments. YM and HT commented on the data and helped to write the manuscript. All authors read and approved the final manuscript.

## Pre-publication history

The pre-publication history for this paper can be accessed here:

http://www.biomedcentral.com/1472-6831/14/110/prepub
